# The first complete mitochondrial genome of *Melicertus* from *M. latisulcatus* (Decapoda: Penaeidae)

**DOI:** 10.1080/23802359.2018.1481792

**Published:** 2018-07-13

**Authors:** Shengping Zhong, Yanfei Zhao, Qin Zhang

**Affiliations:** Key Laboratory of Marine Biotechnology, Guangxi Institute of Oceanology, Beihai, China

**Keywords:** Mitochondrial genome, Melicertus latisulcatus, Decapoda

## Abstract

The western king prawn, *Melicertus latisulcatus*, is a tropical species of ‘grooved shrimps’, which has long adrostral groove along almost the entire dorsal carapace in Penaeidae family. However, the taxonomic revision studies of Penaeidae have been one of the most controversial issues in recent years. In this study, we report the first complete mitochondrial genome of *Melicertus* from *M. latisulcatus*. The mitogenome has 15,971 base pairs (64.7% A + T content) and made up of total of 37 genes (13 protein coding, 22 transfer RNAs, and 2 ribosomal RNAs), and a control region. This study was the first available complete mitogenomes of *Melicertus* and will provide useful genetic information for future phylogenetic and taxonomic classification of Penaeidae.

Penaeidae is a diverse group of economically important marine shrimps, which traditionally been considered to be a primitive group of decapod crustaceans(Lavery et al. 2004). The western king prawn, *M. latisulcatus*, is a predominantly tropical species of Penaeidae throughout the Indo-west Pacific which inhabits open sea areas with sandy or muddy and sandy bottom (Rodgers et al. 2013). There are about 30 extant species in Penaeidae, however, there is still considerable doubt about the taxonomic revision and phylogenetic relationships in this group (Ma et al., 2011). The complete mitochondrial genome is useful molecular techniques for solving taxonomic problems, however, in spite of its economically and ecological importance, adequate Mitogenome information about Penaeidae is still missing (Ma et al. 2009). Here, we report the first complete mitochondrial genome sequence of *Melicertus*, which will provide a better insight into taxonomic classification and phylogenetic relationship of Penaeidae that is the most commercially important decapods worldwide.

A tissue samples of *M. latisulcatus* from three individuals were collected from GuangXi province, China (Beihai, 21.404211 N, 109.263361 E), and the whole body specimen (#GQ0127) were deposited at Marine biological Herbarium, Guangxi Institute of Oceanology, Beihai, China. The total genomic DNA was extracted from the muscle of the specimens using an SQ Tissue DNA Kit (OMEGA, Guangzhou, China) following the manufacturer’s protocol. DNA libraries (350 bp insert) were constructed with the TruSeq NanoTM kit (Illumina, San Diego, CA) and were sequenced (2 × 150 bp paired-end) using HiSeq platform at Novogene Company, China. Mitogenome assembly was performed by MITObim (Hahn et al. 2013). complete mitogenome of *Marsupenaeus japonicus* (GenBank accession number: NC_007010) was chosen as the initial reference sequence for MITObim assembly. Gene annotation was performed by MITOS (Bernt et al. 2013).

The complete mitogenome of *M. latisulcatus* was 15,971 bp in length (GenBank accession number: MG821353), and containing the typical set of 13 protein-coding, 22 tRNA and 2 rRNA genes, and a putative control region. The overall base composition of the mitogenome was estimated to be A 31.1%, T 33.6%, C 20.8% and G 14.4%, with a high A + T content of 64.7%, which is similar, but slightly lower than *Marsupenaeus japonicus* (66.4%) (Zhong et al. 2018). *Melicertus* and *Marsupenaeus* shared very similar morphological characters, which has long adrostral groove along almost the entire dorsal carapace and bears short but distinct gastrofrontal groove behind the eye(Ma et al. 2011). The result of phylogenetic tree of 13 species (including other 12 species from Penaeidae in NCBI) also indicated the close relationship between *Melicertus* and *Marsupenaeus* (Figure 1), which is consistent with the phylogenetic analyses of Penaeidae using combined sequence data of mitochondrial and nuclear genes(Ma et al. 2011). Our mitogenome data supported the sister relationship of *Melicertus* and *Marsupenaeus*. The complete mitochondrial genome sequence of *M. latisulcatus* was the first sequenced mitogenome in *Melicertus*, which will contribute to further phylogenetic and comparative mitogenome studies of *Melicertus*, and related families.

**Figure 1.**
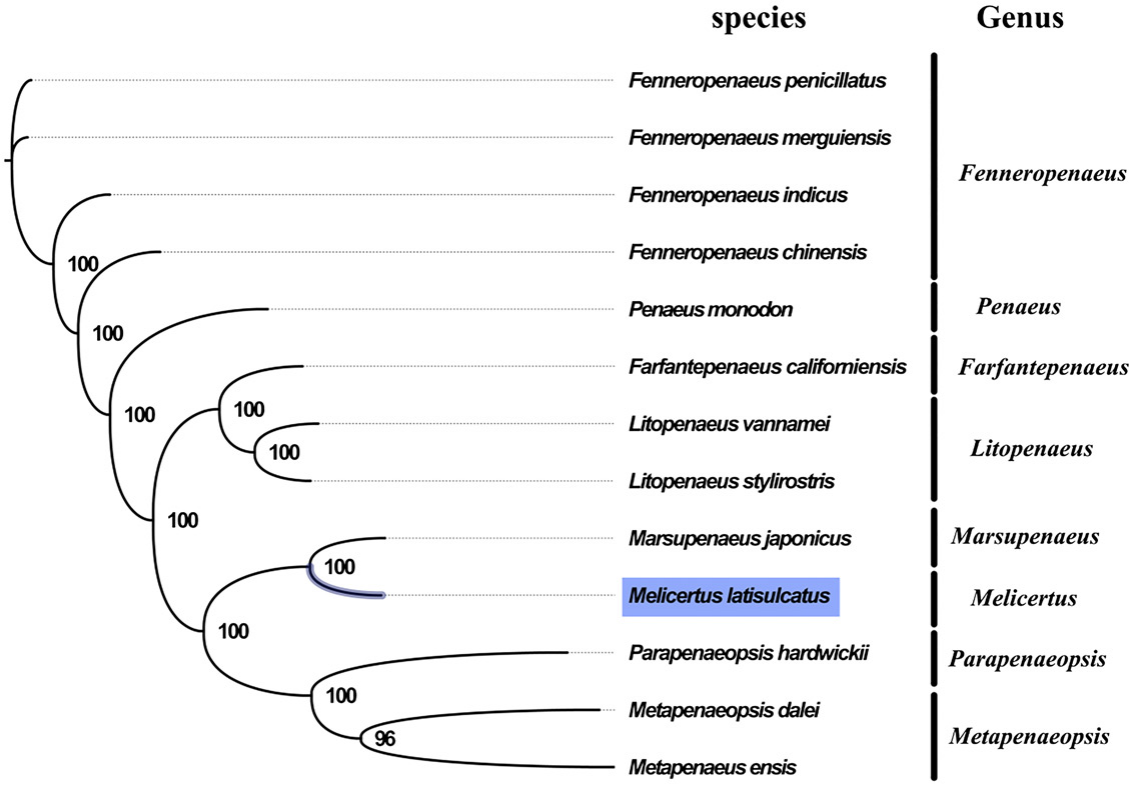
Phylogenetic tree of 13 species in family Penaeidae. The complete mitogenomes is downloaded from GenBank and the phylogenic tree is constructed by maximum-likelihood method with 100 bootstrap replicates. The bootstrap values were labeled at each branch nodes. The gene's accession number for tree construction is listed as follows: *Fenneropenaeus penicillatus* (NC_026885), *Fenneropenaeus merguiensis* (NC_026884), *Fenneropenaeus indicus* (NC_031366), *Fenneropenaeus chinensis* (NC_009679), *Penaeus monodon* (NC_002184), *Farfantepenaeus californiensis* (NC_012738), *Litopenaeus vannamei* (NC_009626), *Litopenaeus stylirostris* (NC_012060), *Marsupenaeus japonicus* (NC_007010), *Parapenaeopsis hardwickii* (NC_030277), *Metapenaeopsis dalei* (NC_029457), and *Metapenaeus ensis* (NC_026834).
